# 

**Published:** 1893-07

**Authors:** 


### CONTENTS.

COMMUNICATIONS.

Things Old, New, and Useful in the Operating Room.................... 313
Report by the Educational Committee on Communications............ 326
The New Dental Law of Ohio............................................ 338
Artificial Crowns....................................................... 342
New Remedies and their Application.................................. 346
Popular Dental Education.............................................. 350
COMMENCEMENTS....................................................... 355

NOTICES.

American Dental Association........................................... 361
The National Association of Dental Faculties—Correction............ 361
Resolutions on the Death of Dr. W. W. Allport......................... 361

EDITORIAL.

Kansas City Dental College............................................ 363
Obituary................................................................ 364

